# Immediate Root-Filling

**Published:** 1891-06

**Authors:** J. A. Libbey

**Affiliations:** Pittsburg, Pa.


### IMMEDIATE ROOT-FILLING.[1]

BY J. A. LIBBEY, D.D.S., PITTSBURG, PA.

At a recent meeting of our local society I gave a clinic on immediate root-filling. There was so much interest manifested in the discussion which followed that I was requested, by a vote of the Society, to read a paper on the subject at this meeting. Whether it was done as a punishment for my deserting the old beaten path, or that I might help some others out of the annoyance so often experienced by continued treatment, "deponent sayeth not."

There is no part of our profession in which I have labored harder than in the treatment of pulpless teeth. The annoyances I have frequently had and the discomfort of the patient has kept me on the lookout for a better mode of treatment. Having tried nearly everything I have heard of, I have finally adopted the following, which I will give in as plain a manner as I can, after which I will cite a few cases from my records for the month of May, which alone are sufficient evidence that it is not so risky as many seem to think.

First, place the rubber dam on the tooth and keep others for convenience, ligate each tooth exposed to view, to be certain of excluding all moisture and prevent alcohol or chloroform from escaping into the mouth, and open into the pulp-chamber so as to get as direct access into canals as possible. With a Swiss jeweller's broach, annealed quite soft, explore the canals, and then with a few fibres of cotton wrapped on the broach clean out the decomposed tooth-pulp. This should be continued until the cotton fibres show very little, if any, moisture. Then use a permanganate of potash solution, one drachm to the ounce, dipping the broach and cotton into it, and introducing it into the canals, repeating this until it shows no discoloration. Second, with an abscess syringe alcohol is injected into the canals; then dry with hot air, repeating once or twice. The best hot-air appliance I have used is the compresssed air-cylinder and accompanying apparatus, demonstrated at a previous meeting of this society by Dr. Register. When thoroughly dried, inject wood-creosote into the canals, either with syringe or broach and cotton, and with a bare broach assist it to the apex. Then inject sufficient chloroform to fill canals, and use

[1] Read before the Pennsylvania State Dental Society, July 29, 1890.

broach again to assist in expelling the air if any should be left in the canal. This is done to exclude the air, and as a carrier for chlora-percha. Before the chloroform has time to evaporate, inject chlora-percha into the canals, and fill the cavity about half full. Then stretch a piece of heavy rubber dam over the cavity, and hold it in this position, and with a ball-burnisher tap at first lightly over the cavity, then increase the force, but not sufficient to puncture the rubber, this will force the chlora-percha into the canals. I am indebted to Dr. Whitesides, of Youngstown, Ohio, for the use of the dam in this manner as well as other hints in this method of root-filling.

With gutta-percha points, previously prepared, of suitable size inserted into the canals, with nerve-canal plugging instrument pack as thoroughly as possible ; then warm a piece of gutta-percha of sufficient size to cover the bottom of the cavity, and use a ball-burnisher vigorously. If the patient shows any sign of pain from the pressure, I desist at that point, feeling satisfied the gutta-percha has reached the apical foramen. I usually fill the cavity with gutta-percha and make an appointment for permanent filling.

If there is a fistulous opening on the gum I try in every case to have the chlora-percha make its appearance through it on the gum.

Now, you will observe I have not classified particular cases. I care not whether it be—

1. Those which are in a healthy condition.[1]

2. Those which are in such a state that slight causes of irritation may excite peridental inflammation.

3. Those of which the peridental membrane is inflamed ; or

4. Those which have been the subject of alveolar abscess, and which are discharging through a fistulous opening.

The following cases, it seems to me, will be sufficient to prove my assertions :

Miss S., aged sixteen ; light hair, light blue eyes ; called latter part of April ; left inferior first molar with large coronal cavity ; pulp almost devitalized ; tooth quite sore to the touch ; extracted the remaining portion of pulp, and treated with wood-creosote on cotton placed loosely in canals, and closed the cavity with cotton saturated with sandarach varnish. The patient returned a few days later with slight soreness ; the treatment was repeated as at first. This treatment was continued, alternating with creosote

---

[1] Classification of Dr. Jack, American System of Dentistry, vol. ii. p. 189.

and iodoform until the fifth treatment. On Friday, May 9, patient returned, and complained of increased soreness to the touch, worse than at any time since first treatment. Up to this time I had not sufficient confidence in immediate root-filling to attempt it with this class,—No. 3. I concluded to fill the canals at this sitting, and filled the cavity with gutta-percha. On Sabbath afternoon I passed her home and saw her sitting on the porch; she called me and said, "I wanted to tell you my tooth is all right, the soreness is all gone!"

Filled permanently June 3.

2. Mr. R., aged about forty; occupation merchant; called May 12; right superior cuspid quite sore to the touch, and upon exploring with a broach in the canal pus flowed out followed by slight bleeding; no fistulous opening. I filled the canal and cavity with gutta-percha. The patient complained of increased irritation that evening, but next morning was much better, since which time there has been no soreness.

3. Miss D., aged eighteen; filled root-canals of right inferior first molar May 13. She had previously been treated five or six times. She was compelled to remove the cotton and sandarach within a few hours, with the exception of the last two treatments. Upon removal of cotton, pus flowed into cavity. This was the condition at this date. No irritation after filling canals.

Filled permanently May 20.

4. Had been treating left superior second bicuspid for Mr. V., aged forty, for the past year, and had closed up temporarily three or four times. Inflammation of the peridental membrane continued. Called May 22 by appointment, and wanted to change his engagement on account of the tooth being sore to the touch, filled root at this sitting as described above. Called July 11; said the last treatment—*i.e.*, May 22—was the best yet. After the next day there had not been any irritation.

5. In the clinic alluded to in the opening of this paper, the patient, a young lady, aged about twenty, the tooth filled at that time was the right superior first molar, and had not had any previous treatment. After the roots were filled, I prepared the cavity on the mesial surface and filled about half full, using hand mallet, with soft gold-foil, No. 4.

I selected this patient for the clinic because on May 17 she came to me with right superior central and both laterals mere shells, and with fistulous openings on the gum. I commenced to open into the canal of the central incisor. I treated with creosote and closed the cavity with cotton, saturated with sandarach, and made an appointment for the 24th. She came back the next day with swollen face. I removed cotton and recommended roasted figs on the gum.

The next week, at the time appointed, I filled all three roots, forcing the chlora-percha through the fistulous openings. May 3 I placed crowns on the three roots. At the time of the clinic, the fourth Thursday of June, all had a healthy appearance: the pink gutta-percha could be seen under the mucous membrane over the left superior lateral, as members here can testify.

